# Macro- and mesoscale pattern interdependencies in complex networks

**DOI:** 10.1098/rsif.2019.0553

**Published:** 2019-10-30

**Authors:** M. J. Palazzi, J. Borge-Holthoefer, C. J. Tessone, A. Solé-Ribalta

**Affiliations:** 1 Internet Interdisciplinary Institute (IN3), Universitat Oberta de Catalunya, Barcelona, Catalonia, Spain; 2 URPP Social Networks, Universität Zürich, Zurich, Switzerland

**Keywords:** complex networks, mesoscale, nestedness, modularity, in-block nestedness, ecological networks

## Abstract

Identifying and explaining the structure of complex networks at different scales has become an important problem across disciplines. At the mesoscale, modular architecture has attracted most of the attention. At the macroscale, other arrangements—e.g. nestedness or core–periphery—have been studied in parallel, but to a much lesser extent. However, empirical evidence increasingly suggests that characterizing a network with a unique pattern typology may be too simplistic, since a system can integrate properties from distinct organizations at different scales. Here, we explore the relationship between some of these different organizational patterns: two at the mesoscale (modularity and in-block nestedness); and one at the macroscale (nestedness). We show experimentally and analytically that nestedness imposes bounds to modularity, with exact analytical results in idealized scenarios. Specifically, we show that nestedness and modularity are interdependent. Furthermore, we analytically evidence that in-block nestedness provides a natural combination between nested and modular networks, taking structural properties of both. Far from a mere theoretical exercise, understanding the boundaries that discriminate each architecture is fundamental, to the extent that modularity and nestedness are known to place heavy dynamical effects on processes, such as species abundances and stability in ecology.

## 1. Introduction

The detection and identification of emergent structural patterns has been a main focus in the development of modern network theory. Such interest is not surprising, because these arrangements lie at the core of the discipline as one of the keys to the origin and dynamics of a network: which assembly rules have led to an observed pattern? How is the system constrained by its structural organization? Those questions, thus, have led to the detection and study of a wide variety of structural patterns, at different scales. Within the ecological literature only, macroscale (i.e. system-wide) patterns such as gradient [1], spatial turnover [2], checkerboard [3,4] and segregation [5] arrangements are prominent examples. At the mesoscale (i.e. regarding groups of nodes), core–periphery [6,7] or combined [1] structures have also attracted the focus of researchers.

**Figure 1.**
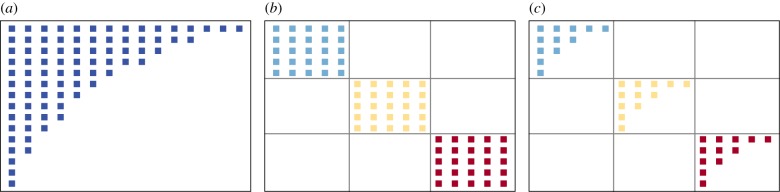
Idealized examples of the structural organization studied in the paper. (*a*) A perfectly nested organization. (*b*) The adjacency matrix of modular network and (*c*) idealized network with in-block nested structure. (Online version in colour.)

But undoubtedly, in the ecological network context, two of these patterns have, by far, concentrated most of the efforts: nestedness and modularity, see figure 1*a*,*b*. Modularity [8–12], a mesoscale pattern [13,14], considers the organization of species as a set of cohesive subgroups, where species within the group interact among them with larger frequency than with species belonging to other groups [15]. Not exclusive to ecological systems, modularity is a widespread organizational structure [16–20]. Nestedness [21,22], a prominent macroscale pattern, quantifies to which extent specialist interacts with a subset of the species or individuals that generalists interact with [23,24]. It stands as a frequent emergent structural arrangement, which has been observed prominently in ecology [21,25], but also in economy [26–28] and social systems [29].

Overall, the knowledge acquired in the last 40 years has unveiled some of the implications each of these individual organizational patterns have on a system’s dynamics. However, we have limited knowledge on how different structural signatures may interlace, or how—if ever—they affect and limit each other. A clear example is the enduring debate around nestedness and modularity, and their possible co-occurrence in a single structure [30,31]. On one side, nested arrangements promote the persistence (i.e. increase in abundances) of mutualistic ecological systems [32–34]; but, at the same time, they minimize the system’s linear stability [33,34]. On the other, modular organization maximizes stability [35,36] and biodiversity [32].

Thus, we should expect abundant, diverse and stable systems to develop jointly both these structural arrangements, in ecology and elsewhere [26,27,29]. So far, however, we lack conclusive evidence that fulfils such expectation: the analysis of real data examples has repeatedly shown that nestedness and modularity may [29–31,37,38] or may not [29] coexist. Furthermore, the combination of both structures in the same network has been also assessed, either as communities with nested structure within (in-block nestedness) [1,37–39] or as communities that organize forming a nested structure [31]. And yet, we are missing a systematic approach that tackles the plausible coexistence of nested and modular patterns.

In this work, we show that purely structural constraints forbid interactions to be completely modular and completely nested at the same time, with room, however, for a wide range of intermediate possibilities. Such mutual constraints offer an explanation to the inconclusiveness of the aforementioned efforts: modular (but not nested), nested (but not modular) and modular–nested networks are all feasible outcomes for a system that is confronted with a multi-objective (abundance versus stability) optimization problem, i.e. without a solution that can simultaneously optimize each objective separately. Thus, our contribution to understanding the conditions for the coexistence of these structural patterns may shed light on the dynamical trade-offs that either arrangement can facilitate. It is worth highlighting that our approach takes into account solely the structural aspect of the problem, without considering a plausible dynamic co-emergence of both patterns [40,41], which we foresee as the next relevant problem along these lines.

The article is organized as follows: §2 provides the necessary definitions for nestedness, modularity and in-block nestedness. Section 3 provides the details for a toy model that enables the construction of a suitable synthetic benchmark, which is fully exploited in §4. Sections 5 and 6 are devoted to the analytical relationship between the three structural patterns at stake. Sections 7 and 8 stretch the analytical findings to provide, for empirical networks, approximate bounds to nestedness and modularity. The discussion in §9 summarizes all the previous, and considers some research lines that stem from the current findings.

## 2. Structural patterns in ecological networks

In this section, we introduce the basic notions that appear throughout the paper. For the sake of simplicity, we only report definitions for unipartite and symmetric networks. The extension to asymmetric and bipartite systems is not difficult but requires more intricate notation, as well as the consideration of the different number of species that may compose each network dimension. These extensions are provided in the electronic supplementary material.

Explanations in the subsequent sections refer to networks, which are often represented in the ecological literature as binary (presence–absence) adjacency matrices *A*, whose elements are *a*_*ij*_ = 1 if species *i* has been observed in location *j*, and zero otherwise (e.g. in biogeography); or whose elements are *a*_*ij*_ = 1 if species *i* interacts with species *j*, regardless of spatial relations (e.g. community ecology). However, in purely structural terms there are no main differences in the structure of these two representations.

### 2.1. Nestedness

The concept of nestedness was introduced to describe the patterns of distribution of species in isolated habitats [23]. Outside ecology, it has been observed in different contexts [26,27,29]. Besides its interest as a descriptor, nestedness has revealed itself as a key pattern in the dynamics of ecosystems [32–34] (and not without debate).

In a perfectly nested network, the set of neighbour nodes (neighbour species) with few interactions (or low degree) are a subset of those with larger degree. (Without loss of generality, in the following we consider purely unipartite networks. The extension to bipartite ones is trivial; figure 1*a*.) From an algebraic perspective, the spectral properties of such perfectly nested graphs have been studied by mathematicians [42–44], which later facilitated the proposal of a robust detection method [45], in which Staniczenko *et al.* quantified nestedness with respect to the maximum eigenvalue of binary and weighted graphs’ adjacency matrices. In a different tradition, ecologists have also dedicated many efforts to quantify nested structures in real systems. In first place, there are measures based on counting misplaced relations to complete a perfect upper triangular nested structure in *A*, such as the nested temperature (NT) measure, introduced by Atmar & Patterson [21]. To overcome some pitfalls around placement-based measures, Almeida-Neto *et al.* [46] developed overlap metrics, such as the node overlap and decreasing fill (NODF), which considers the amount of common neighbours between every two pair of nodes in matrix *A*, alongside with its weighted version [47–49]. In this work, we stick to a NODF-like descriptor since it allows much easier analytical development.

For the case of a unipartite symmetric system with *N*_*T*_ species encoded in matrix $A\in {\left\{1,0\right\}}^{N_{T}\times N_{T}}$ and *a*_*ij*_ = *a*_*ji*_, nestedness can be measured as

2.1 $$N=\frac{2}{N_{T}}\left\{\sum_{ij}^{N_{T}}\left[\frac{O_{ij}-\langle O_{ij}\rangle }{k_{\;j}(N_{T}-1)}\Theta \;(k_{i}-k_{\;j})\right]\right\},$$

where $O_{ij}=\sum_{k}a_{ik}a_{jk}$ accounts for the amount of commonly shared neighbours between species *i* and *j* (a.k.a. overlap); $k_{i}=\sum_{k}a_{ik}$ corresponds to the degree of node *i* and quantifies the number of species with whom *i* is related to; and *Θ* ( · ) is the Heaviside step function that ensures that *O*_*ij*_ has a positive contribution when *k*_*i*_ ≥ *k*_*j*_. Additionally, *O*_*ij*_ is conveniently corrected by a null model that discounts the expected change of each species have to share a neighbour [39], namely the expected overlap $\left\langle O_{ij}\right\rangle$. Assuming no correlation between neighbouring species of *i* and *j* the probability of sharing a particular neighbour only depends on the degree of *i* and *j* and on size of the network, $(k_{i}k_{\;j})/N_{T}^{2}$. Hence, the average overlap is $\langle O_{ij}\rangle =\sum_{k=1}^{N_{T}}(k_{i}k_{\;j})/N_{T}^{2}=(k_{i}k_{\;j})/N_{T}$. The presence of a null model term enforces N∈[0,1). Note that equation (2.1) follows NODF closely (and often reduces exactly) (Almeida-Neto *et al.*) if the term $\left\langle O_{ij}\right\rangle$ is suppressed.

### 2.2. Modularity

Modular structure is a rather ubiquitous mesoscale architecture [16,17,19,20]. It comes under many names (communities, compartments, modules, clusters), which amount to the fact that nodes are organized to form groups, i.e. devoting many links to nodes in the same group, and fewer links towards nodes outside [15] (figure 1*b*). This simple intuition hides behind an NP problem, provided that the number of possible ways to partition a graph scales faster than polynomial with respect to the network size: even for really small graphs, an exhaustive assessment of every partition’s fitness becomes unfeasible. For this reason, the network science community has developed a rich collection of algorithms and methodologies to infer these communities from relational data. Probably, the most popular method in network science, and in particular in ecology, is through the maximization of a fitness function called modularity *Q* ∈ [0, 1) [13]. We opt for this definition of communities because, besides being the most popular, it allows for the set of analytical developments in §§5 and 6. Formally,

2.2 $$Q=\underset{\alpha }{\text{argmax}}\frac{1}{2L}\sum_{i,j=1}^{N_{T}}\left(a_{ij}-\frac{k_{i}k_{\;j}}{2L}\right)\delta (\alpha_{i},\alpha_{\;j}),$$

where *L* is the total number of links in the network, ***α*** is a vector representing the membership variable of species with entries *α*_*i*_, *δ*(*α*_*i*_, *α*_*j*_) corresponds to the Kronecker delta, equal to one if nodes *i* and *j* belong to the same community (zero otherwise) and *k*_*i*_ *k*_*j*_/2*L* is a regularization term to discount the expectation that two nodes are connected by chance considering the degree they have. Furthermore, the original fitness function can be rewritten in terms of the total contribution per community as

2.3 $$Q=\underset{\alpha }{\text{argmax}}\sum_{c=1}^{B}\left[\frac{l_{c}}{L}-{\left(\frac{d_{c}}{2L}\right)}^{2}\right],$$

where *B* is the number of communities, *l*_*c*_ is the total number of links in community *c*, and *d*_*c*_ is the sum of the degrees of all nodes in such community. To ease readability, there are no explicit membership variables *α* in equation (2.3), which are implicit in variables *d*_*c*_ and *l*_*c*_.

The actual algorithms to find a (sub)optimal partition come under different flavours, i.e. considering the dynamical properties of the network at stake [50], probabilistic inference [51], etc. See [52] for an extensive review. Furthermore, equations (2.2) and (2.3) can be adapted to account for the underlying nature of the network (weighted [10], signed [53], multilayer [54], etc.). Of special interest for ecology, Barber modularity [12] addresses the specificities of bipartite networks, in which relations between pairs of species in the same set or guild are forbidden. In this case, the regularization term equation (2.3) is modified accordingly.

### 2.3. In-block nestedness

The existence of concurrent nested and modular organizations in networks has been debated in different contexts [29–31]. A step beyond mere concurrence, we find the possibility of combined architectures [1,37–39], i.e. networks with a modular layout, where interactions within each module (or block) are nested (figure 1*c*).

The original approaches [1,37,38] to detect nested compartments started by first optimizing modularity, then subsequently computing the level of nestedness exclusively for nodes within the detected communities. Although this sequential procedure delivers good results in many situations (as detected modules often gather nodes with degree heterogeneity [55]), a specialized fitness function is required for the general case. Thus, as in the case of modularity, in-block nestedness detection is a hard computational problem where the use of heuristic algorithms is mandatory. Using the formulation developed in [39], the degree of in-block nestedness of a network I can be computed as

2.4 $$I=\frac{2}{N_{T}}\left\{\sum_{i,j}^{N_{T}}\left[\frac{O_{ij}-\langle O_{ij}\rangle }{k_{\;j}(C_{i}-1)}\Theta \;(k_{i}-k_{\;j})\delta \;(\alpha_{i},\alpha_{\;j})\right]\right\},$$

where *C*_*i*_ is the size of the community to which node *i* belongs and as in the two previous cases, I∈[0,1). It is worth highlighting that this hybrid structure reframes nestedness, originally a macroscale feature, to the mesoscopic level. In this sense, a perfectly nested structure corresponds to an in-block nested structure with a single community, i.e. equation (2.4) reduces *exactly* to equation (2.1) when *B* = 1.

Other methodologies that may detect communities with similar structural arrangements within modules exist, e.g. core–periphery [56], but there is no guarantee that the detected communities have nested properties.

## 3. Synthetic network generation model

We start the analysis of the trade-offs between the different patterns of interest (nestedness, modularity and in-block nestedness) by exploring an extensive, controlled synthetic setting. To this aim, we have extended a network generative model, recently proposed in [39], to generate networks with a fixed block size and increasing number of blocks (hence, increasing network size), instead of networks with a fixed size. The model assumes statistical independence between the existence of species interactions and pivots on four parameters: the number of communities *B* ∈ [1, ∞), noise regarding the existence of interactions outside species communities *μ* ∈ [0, 1], noise regarding interactions outside a perfectly nested structure *p* ∈ [0, 1] and a shape parameter for the generation of the nested structure *ξ* ∈ [1, ∞]. The shape parameter controls the slimness of the nested structure. Although *ξ* affects the overall network connectance (total number of existing species interactions), it does not determine it: for example, for a network with a single block (*B* = 1) and *ξ* = 1, the matrix fill is 50%. For the same *ξ* = 1, with *B* = 2, the fill is 25%. On the other hand, *p* and *μ* do not alter the density of the network. We refer the reader to [39] for a formal proof of this aspect.

**Figure 2.**
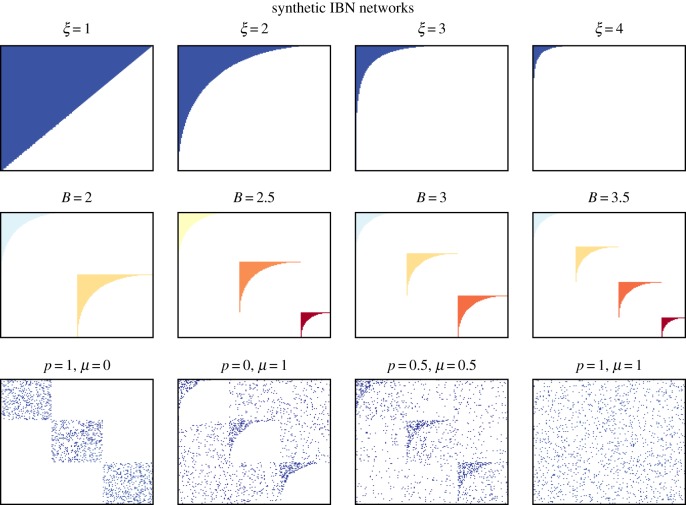
Examples of synthetic network generation with the model introduced in [39]. The top and middle rows show the effects of the shape parameter *ξ* and the number of blocks *B*, respectively, in a noiseless scenario (*p* = *μ* = 0). The bottom row provides some examples of the effect of the noise parameters *p* and *μ*. (Online version in colour.)

Figure 2 shows some of the synthetic networks that the model is able to generate. The first row of the figure shows perfectly nested networks generated with *B* = 1, varying values of *ξ* and fixing *p* = *μ* = 0. The second row shows perfect in-block nested networks obtained with the same settings *B* > 1 and *p* = *μ* = 0. Finally, the third row shows intermediate scenarios varying *p* and *μ*. The left example shows an ideal modular network (in terms of modularity), i.e. with no links between communities (bottom-left); and the right example is a purely random Erdős–Rényi network, regardless of *B* (bottom-right).

Our procedure enables generation of networks with fractional communities, in the sense that the integer part of *B* (⌊B⌋) controls the number of blocks of equal size, and the decimal part determines the relative size of a remaining block with respect to the others. Specifically, we construct the adjacency matrix of a network by building ⌊B⌋ blocks of size *N*_*B*_ and a remaining block of size $(B-\lfloor B\rfloor )N_{B}$, forming a block diagonal matrix. The choice of generating fractional communities allows for a smoother transition between networks of different integer community sizes (*B* = 1, 2, 3, 4, …), which, in turn, contributes to generate a finer synthetic evaluation.

Considering all the above-described parameters, we can derive the independent probability expressions for having an interaction between species *i* and *j* within community *c* as

3.1 $$\begin{array}{rl} P(A_{ij}^{c}) & =[(1-p+pp_{r})\Theta \;(jN_{B}-f_{n}(iN_{B}))\\ & \;+p_{r}(1-\Theta \;(jN_{B}-f_{n}(iN_{B}))]\;(1-p_{\mu }), \end{array}$$

where the term *f*_*n*_ corresponds to the *p*-norm ball curve, drawn for a given *ξ* value, and employed to generate the perfect nested structure. Equation (3.1) implicitly models a two-step process:

— (*T*1) by which we first remove from the ideal nested structure the interactions that will be considered as noise, and then,
— (*T*2) these interactions are randomly distributed over the set of remaining non-existing interactions.

The term within square brackets differentiates between the probability of having an interaction within the nested part and outside the nested part inside the community. These two components are separated by the Heaviside function *Θ*. Having an interaction within the nested part implies either the interaction has not been removed in *T*1, (1 − *p*), or that has been removed in *T*1 then recovered in *T*2, *pp*_*r*_. The probability of recovering the interaction *p*_*r*_ is proportional to the number of interactions that have been removed in *T*1, *pL* and inversely proportional to the number of non-existing interactions in the network, *N*_*B*_ − *L* + *pL*. That is, *p*_*r*_ = *pL*(*N*_*B*_ − *L* + *pL*)^−1^, where *L* is the total number of interactions within the network. The rest of equation (3.1), *p*_*r*_ corresponds to the probability of having a link outside the initially nested part. Finally, the term $\left(1-p_{\mu }\right)$ stands for the probability of not removing the link in the process of generating inter-block noise and $p_{\mu }=\mu (B-1)/B$. Finally, the probability of an inter-block link, between species *i* and *j* belonging to different communities is given by

3.2 $$P(A_{ij}^{o})=\frac{2Lp_{\mu }}{2(B-1)N_{B}^{2}}=\frac{\mu L}{N_{B}^{2}B},$$

where the numerator corresponds to the number of removed interactions within communities in *T*1, and the denominator corresponds to the possible places where each of those links can be relocated in step *T*2.

The corresponding software codes to generate synthetic networks with nested, modular and in-block nested structures, can be downloaded from the web page of the group http://cosin3.rdi.uoc.edu/, under the Resources section.

## 4. Structural analysis of synthetic networks

We begin the study of the interdependence between the different structural measures (N, *Q* and I) with the analysis of synthetic structures constructed with the model presented in §3. We generated a set of 2 × 10^5^ networks with varying parameters, using equations (3.1) and (3.2). To be precise, we cover the following parameter ranges: *B* ∈ [1, 9]; *ξ* ∈ [1.5, 7]; *p* ∈ [0, 0.6]; and *μ* ∈ [0, 0.6]. We restrict *p* and *μ* to 0.6 to guarantee that some identifiable pattern is still present, e.g. maintaining the requirements of weak community structure as defined in [15], while avoiding spurious outcomes [57]. See section V of the electronic supplementary material for detailed information. We have assumed a fixed community size of *N*_*B*_ = 50. Thus, as we add communities, network size increases proportional to *B* parameter. The alternative process of fixing *N*_*T*_ and reducing the size of the communities as we increase *B* produces equivalent results, but is difficult in the analytical approach of the following sections. For modularity and in-block nestedness maximization, we have used the extremal optimization algorithm [8], adapted to the corresponding objective functions (equations (2.3) and (2.4), respectively). The corresponding software codes, both for uni- and bipartite cases, can be downloaded from the web page of the group http://cosin3.rdi.uoc.edu/, under the Resources section.

**Figure 3.**
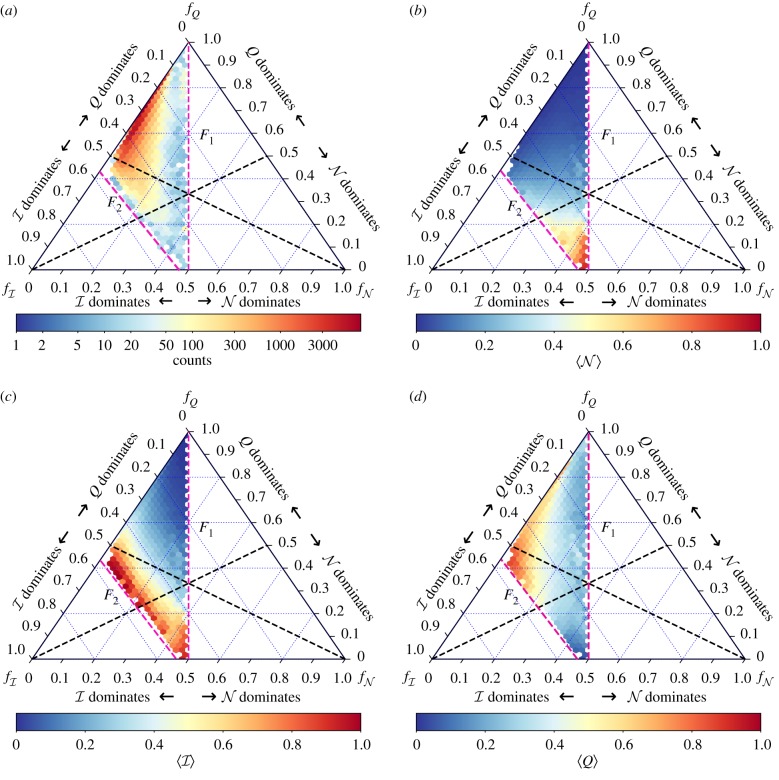
Ternary plots showing the joint influence of the different structural patterns analysed within the paper. Each axis corresponds to the fractional values of the three structural patterns, i.e. $f_{N}=(N/N+Q+I)$, $f_{Q}=(Q/N+Q+I)$ and $f_{I}=(I/N+Q+I)$. The bottom axis represents N, and its right vertex corresponds to perfectly nested networks ($f_{N}=1$). Other values of $f_{N}$ are indicated by the dashed blue lines in the direction ↗ of the triangle. The right axis represents *f*_*Q*_, and the top vertex thus corresponds to purely modular networks (*f*_*Q*_ = 1). Other *f*_*Q*_ values are indicated by horizontal dashed blue lines. Finally, the left axis represents $f_{I}$, and the left vertex corresponds to networks that are purely in-block nested ($f_{I}=1$). Other $f_{I}$ values are indicated by lines in the direction ↘ of the triangle. Additionally, the black dashed lines delimit dominance regions. Each dominance region indicates (by pairs) which is the dominating structural pattern. For the sake of clarity, the dominant structure is also indicated close to the plot axis. We refer the reader to the electronic supplementary material for further details on the construction and interpretation of ternary plots. (*a*) The distribution of the generated networks over the ternary plot. The colour bar indicates the amounts of networks in each bin. (*b*–*d*) The average values of N, I and *Q*, respectively. (Online version in colour.)

Figure 3 presents the results over four ternary heat-map plots. This is a convenient diagram, since it allows the joint assessment of the mutual relationships between the three different structural patterns under consideration. Figure 3*a* shows a density plot, showing the structural properties of the generated networks. The colour indicates the amount of networks in each bin of the ternary plot. As we see, the network generation model does not produce structures homogeneously distributed over all the domain. It is apparent that the predominant architecture is modular. This is expected, since any parameter configuration with *B* > 1 (more than 95% of the generated benchmark) presents some sort of community organization. Furthermore, modularity is the most favoured arrangement among the three under discussion: any departure from *B* = 1, and any departure from *p* = 0, decreases nestedness and in-block nestedness, but leaves *Q* unmodified. In other words, only parameter *μ* affects modularity in a negative way. However, this bias in the generation process does not affect our conclusions since we ensured that expected values for each hexagonal bin presented on the rest of the paper contains a minimum sample size of 20 networks. An alternative way to overcome this sampling problem in the non-homogeneous space may be to apply stratified sampling techniques.

Figure 3*b*–*d* of the same figure reports the average value of N, I and *Q* in each hexagonal bin of the ternary. A preliminary visual analysis shows that the highest values of N and *Q* never overlap (red areas in Figure 3*b*,*d*). By contrast, I is able to maintain high values for networks that are either modular or nested. These are valuable insights for the analytical results in the remainder of the article: they numerically confirm that networks cannot acquire properties of nestedness and modularity simultaneously, whereas in-block nested networks might.

Another remarkable feature of the results in figure 3 is the existence of sharp boundaries in the ternary plots, conveniently marked in dashed red. The first boundary, *F*_1_, results from the definition of I, which generalizes N. As stated above, equation (2.4) reduces to equation (2.1) when *B* = 1. Translated to coordinates on the ternary, *F*_1_ simply reflects that the contribution of N is always equal to or smaller than the contribution of I. Thus, this holds also in fractions $f_{N}\leq f_{I}$. More interesting, however, is the existence of *F*_2_, which suggests that there is an inherent limit that constrains in-block nestedness to dominate over *Q*. On close inspection (see figure S2 of the electronic supplementary material), networks which map onto *F*_2_ have high values of *ξ*, and very low values of *p* and *μ*. We build on this observation to elaborate our analytical exploration below.

## 5. Structural analysis for a ring of star graphs

**Figure 4.**
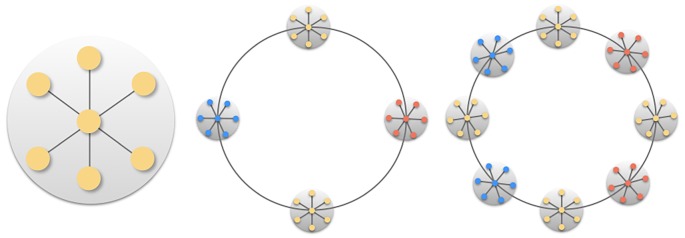
Design of a ring of star graphs. The star graphs are connected through their central nodes. (Online version in colour.)

In this section, we derive analytically the expressions for N, I and *Q* at the boundary *F*_2_ represented in figure 3. This is possible because, as mentioned, networks that map onto that boundary have common and very specific features: an extreme fill parameter, *ξ* → ∞ (i.e. very sparse network), a perfectly nested intra-block structure (*p* = 0), and minimum inter-block connectivity (*μ* ≈ 0); see figure S2 of the electronic supplementary material. Such organization corresponds exactly to a well-defined family of network configurations: a ring of star graphs, G* hereafter that reduces to a single star when *B* = 1, and resembles a set of stars connected with a single link through their central nodes when *B* > 1 (so as to guarantee a single giant component) (figure 4).

The exact expressions for N, I and *Q* for G* are the key to understand the mutual constraints that the different network arrangements impose on each other, strictly for such an idealized case, and more loosely in general. In the following, we consider a ring of star graphs with *B* communities and *N*_*B*_ nodes per community. For such given graph, we find the exact values $N_{G^{*}}$, $I_{G^{*}}$ and *Q*_G*_. The steps behind each expression presuppose unipartite networks. We are aware that, in ecology and elsewhere, bipartite networks are more prominent when it comes to study nestedness, but the required notation and length of the equations are much less manageable. Thus, below we restrict for the unipartite case and we provide the analytical values for the bipartite case in the electronic supplementary material.

### 5.1. Nestedness

We derive the analytical expression for $N_{G^{*}}$ from the expression in equation (2.1). The pair overlap of a generalist node (the centre of each star subgraph), *g*, with a specialist node (periphery of a star), *s*, is $O_{gs}/k_{s}=1$ if *g* and *s* belong to the same star (and 0 otherwise). For all those pairs (regardless of the star they belong to), the null model contribution is $\langle O_{gs}/k_{s}\rangle =(N_{B}+1)/BN_{B}$. We can obtain in a similar way the terms for the generalist–generalist pairs between stars. Summing up all the contributions, the final expression for $N_{G^{*}}$ is as follows:

5.1 $$N_{G^{*}}=\frac{BN_{B}^{3}-BN_{B}^{2}-3BN_{B}+B+2N_{B}+2}{BN_{B}\;(BN_{B}^{2}+BN_{B}-N_{B}-1)}.$$

The corresponding expression for bipartite networks can be obtained following a similar logic, but taking into consideration the contributions of rows and columns separately (*N*_r_ and *N*_c_). This consideration renders a cumbersome equation which impedes the readability of the present section. Hence, the corresponding expression, along with its complete derivation, is available in section IIIA of the electronic supplementary material.

### 5.2. Modularity

In general, the optimal partition for an arbitrary network cannot be easily obtained, except for very idealized cases such as G*, where each star in the ring forms a community (note that G* does not suffer, like a ring of cliques, from the well-known *Q*’s resolution limit [58]).

On such a setting, we can easily derive the contribution of each star to the total *Q* following equation (2.3). The total number of links within communities is *l*_*c*_ = *N*_*B*_ − 1 and the amount of links of the network, including links within and between communities, is *L* = *B*(*N*_*B*_ − 1) + *B* = *BN*_*B*_. The last term, the sum of the degrees of all the nodes in community *c*, corresponds to *d*_*c*_ = 2*N*_*B*_. Assembling these, we obtain the modularity of G* as

5.2 $$Q_{G^{*}}=B\left[\frac{N_{B}-1}{BN_{B}}-{\left(\frac{2N_{B}}{2BN_{B}}\right)}^{2}\right]=1-\frac{1}{N_{B}}-\frac{1}{B},$$

which is equivalent to the general expression derived in [58]. The bipartite counterpart of equation (5.2) and its complete derivation is available in section III-B of the electronic supplementary material.

### 5.3. In-block nestedness

The derivation of I_G*_ resembles that of N_G*_, with the difference that only nodes within the same community contribute; thus, all stars have the same contribution. Focusing now on each star, we have only two contributing terms to the sum: the pair overlap between specialist nodes, *s*, and the pair overlap of the generalist node, *g*, with the specialists. In both cases, the contribution is 1. The null model corrections are $\langle O_{gs}\rangle =k_{g}k_{s}/BN_{B}=(N_{B}+1)/BN_{B}$ and $\langle O_{ss}\rangle =k_{s}k_{s}/BN_{B}=1/BN_{B}$. Finally, the size of the communities is *C*_*g*_ = *C*_*s*_ = *N*_*B*_. Replacing all the contributions in equation (2.4), we obtain

5.3 $$I_{G^{*}}=1-\frac{3}{BN_{B}}-\frac{2}{N_{B}}.$$

The complete derivation of equation (5.3) for the bipartite case is also available in section III-C of the electronic supplementary material.

The expressions presented above consider a closed ring, on which the number of inter-community links is *B*. For the cases *B* = 1 and *B* = 2, the number of inter-community links is *B* − 1 and the degree of the generalist nodes is *k*_*g*_ = *N*_*B*_ − 1 and *k*_*g*_ = *N*_*B*_, respectively. Thus, the expressions for these particular settings demand a specific treatment, see section II of the electronic supplementary material for details on these cases.

## 6. Exact constraints between N_G*_ and *Q*_G*_

The interdependences of equations (5.1), (5.2) and (5.3) become apparent when the number of blocks, *B*, or the size of the blocks, *N*_*B*_, are large. For the case where *N*_*B*_ → ∞, equations (5.1), (5.2) and (5.3) reduce to

6.1 $$\begin{array}{rl} & \lim_{N_{B}\to \infty }N_{G^{*}}=\frac{1}{B},\;\lim_{N_{B}\to \infty }Q_{G^{*}}=1-\frac{1}{B}=1-N_{G^{*}},\\ & \;\lim_{N_{B}\to \infty }I_{G^{*}}=1. \end{array}$$

As we see, for large G* networks, nestedness and modularity are complementary corroborating the empirical observations in [29]. This result shows analytically that, in large systems with low fill, the ecosystem needs to choose (with the dynamical consequences it may bear) between maximizing community structure, or maximizing nested arrangements, but not both at the same time.

With respect to the case *B* → ∞, equations (5.1), (5.2) and (5.3) reduce to

6.2 $$\begin{array}{rl} & \lim_{B\to \infty }N_{G^{*}}=0,\;\lim_{B\to \infty }Q_{G^{*}}=\frac{N_{B}-1}{N_{B}}\approx 1,\\ & \;\lim_{B\to \infty }I_{G^{*}}=\frac{N_{B}-2}{N_{B}}\approx 1. \end{array}$$

In this case, the existence of many communities implies the impossibility to develop a purely nested pattern. Indeed, the mutual bounds that N_G*_ and *Q*_G*_ impose on each other are evident, displaying a perfect anti-correlated behaviour. A plausible way to preserve both nested arrangements and community structure is under the form of in-block nestedness, which yields to the maximum possible value in both limits. Importantly, this suggests that I does not show any incompatibility with either N or *Q*.

**Figure 5.**
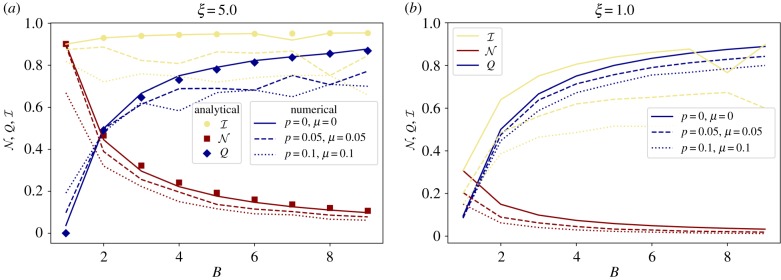
(*a*) Comparison of the analytical (symbols; equations (5.1)–(5.3)) and numerical (lines) values of N, *Q*, I with respect to *B*. All the calculations were performed by taking *N*_*B*_ = 50 and *ξ* = 5. The values for *p* and *μ* parameters, which increasingly depart from the ideal configuration G*, are indicated in the plot legend. (*b*) Same exercise as (*a*), for a very dense network (*ξ* = 1). The overall behaviour of N and *Q* is preserved, with monotonic decrease and increase, respectively. I is closer to N initially, but quickly converges to *Q* thereon. Notably, the analytical N–*Q* antagonism does not hold anymore for low *B*, as these networks deviate strongly from G*. (Online version in colour.)

Figure 5*a* illustrates these results, comparing the analytical estimation of N_G*_, *Q*_G*_ and I_G*_ (equations (5.1)–(5.3)) against *B*, and the numerical results for networks generated from different parameters. As the generated networks deviate from the ring of stars G* (i.e. *p* > 0 and *μ* > 0), results show a worse fit to the analytical prediction, but the overall anti-correlated pattern clearly remains. Finally, we observe that as networks transition from a nested (*B* = 1) to a modular (*B* > 1) architecture, the values of in-block nestedness remain very high (close to one as equations (6.1) and (6.2) indicate) and almost constant. Figure 5*b* tests the same evolution for a much denser network (50% of matrix fill when *B* = 1, clearly far above most real networks). The anti-correlated behaviour of N and *Q* is preserved, but the effects of the null model term are notable: the maximum value that nestedness can take (at *B* = 1) is N≈0.3.

## 7. Approximate constraints N and *Q* (general case)

Results in §6 obtained for idealized settings (G*) point to a more general question: can the exact constraints in equations (6.1) and (6.2) be used to understand the co-occurrence of macro- and mesoscale patterns for the general case? Can we exploit the complementarity between N and *Q* beyond the strict conditions of G*? This and next section target these questions, proposing soft bounds for *Q* (and for I) in terms of N when networks deviate from idealized scenarios. We stress the importance of this attempt since N can be obtained for any network in polynomial time, $O(N_{T}^{3})$, while the maximization of *Q* and I are NP problems. In this situation, these bounds offer a valuable *a priori* intuition of the mesoscale organization of a network. The derivation of these bounds for an arbitrary network *G* is presented below.

### 7.1. Upper bound

The calculation of N is computationally cheap even for very large networks. Thus, given N for a graph *G*, to obtain the maximum *Q* value compatible with this level of nestedness, we assume *G* can be approximated to G* with the same number of nodes, *N*_*T*_, and nestedness N. That is, *G* is assumed to have a relatively large *ξ*. The rationale behind this mapping (*G* to G*) responds to the fact that, for any network with the given N, the largest possible modularity value corresponds to a network lying on *F*_2_, i.e. the G* graph (figure 3*d*). With this approximation, the upper bound reduces to computing *Q*_G*_ (equation (5.2)) and I_G*_ (equation (5.3)) for a G* network compatible with the observed values of N_G*_ (equations (5.1)). To attain these, the only missing information is the number of communities, *B*, which, for the case of G* with equally sized modules (that is, *N*_*T*_ = *BN*_*B*_), can be obtained exploiting equation (5.1):

7.1 $$N_{G^{*}}(N_{T},B)=\frac{\left(B^{3}+B^{2}(2-3N_{T})-B(N_{T}-2)N_{T}+N_{T}^{3}\right)}{B(N_{T}-1)N_{T}(B+N_{T})}.$$

This polynomial equation has three possible roots, two of them being in the imaginary domain. The upper bounds for *Q* and I are thus readily available, applying *B* to equations (5.2) and (5.3). We remark that this is a heuristic approximation to *Q* upper bound. The consideration of a more nuanced estimation, which should consider the density of *G* and non-homogeneous communities, is beyond the purpose of this work. Additionally, we can obtain the fractional contributions of *Q*, I and N over the *F*_2_ boundary, $f_{Q}^{F_{2}}$, $f_{I}^{F_{2}}$, $f_{N}^{F_{2}}$ (figure 3). In particular, $f_{Q}^{F_{2}}$ will be required to estimate the lower bound.

### 7.2. Lower bound

We now turn our attention to the minimum value that *Q* can attain, which is obtained at the boundary *F*_1_, see figure 3*d*. Heuristically, this makes sense because networks which belong to the region along *F*_1_ are those with *B* = 1, see electronic supplementary material, figure S2*a*. To obtain the lower bounds for *Q* we assume that N values are approximately constant with respect to the contributions *f*_*Q*_. This is not a strict fact, but an observation from figure 3*b*. Additionally, at the boundary *F*_1_, we know that N=I. Thus, with the actual measure of N, and $f_{Q}^{F_{2}}$ obtained though the upper bound estimation, we can obtain *Q* as

7.2 $$f_{Q}^{F_{1}}\approx f_{Q}^{F_{2}}=\frac{Q}{Q+I+N}=\frac{Q}{Q+2N}.$$

The lower bound for I does not need a heuristic estimation because, as mentioned, its definition implies a hard lower limit when *B* = 1, i.e. I=N.

**Figure 6.**
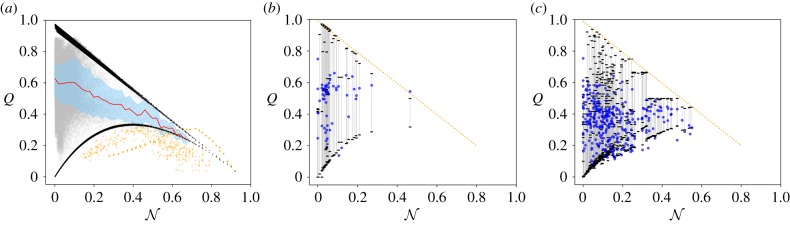
(*a*) The values of *Q* obtained after optimization (grey and yellow dots), plotted against N for over 2 × 10^5^ generated networks. Yellow points correspond to networks with *B* = 1 (network with a single block), for which modularity optimization algorithms detect more than one block, although only one planted block exists in the network. The red line in (*a*) indicates the average values of *Q* for a given N, the error shaded area represents one standard deviation above and below that average; networks with *B* = 1 are excluded in this computation. (*b*) Results obtained for the set of 57 unipartite social network analysed in [39]; and (*c*) for the set of bipartite social and ecological networks [59,60]. In these two panels, dark blue dots represent the real *Q* value after optimization, and bars represent the corresponding estimated upper and lower bounds for the same network. In all scenarios, the upper and lower bounds of *Q* are marked by black dots. (Online version in colour.)

Figure 6*a* shows the values of *Q* as a function of N for the previous synthetic ensemble (∼2 × 10^5^ networks). *Q* values, as obtained with the optimization algorithm, are plotted in grey or yellow, and the values of the theoretical upper and lower bounds are plotted in black. The red line indicates the average values of *Q* for a fixed N, and the overlaid error area represents one standard deviation above and below that average. Our approximation of *Q* bounds is in good agreement with actual values obtained after optimization: most of the optimized *Q* values lie within the estimated soft bounds. Despite the wide range of parameters *ξ*, *B*, *p* and *μ*—far from limiting cases in most cases—estimated upper bounds behave like Q=1−N almost perfectly, in accordance with our analytical insights. While these bounds are trivial when N≈0, we observe that, for intermediate-to-high values of nestedness, these provide relevant information about the possible mesoscale organization of the network.

Modularity values, *Q*, above the upper bound correspond to networks with a single community *B* = 1 and perfectly nested structure, *p* = 0 (see electronic supplementary material, figure S3). These networks, coloured as yellow in figure 6*a* and less than 0.1% of the total, are dense enough to allow a partition with *B* > 1 where the nodes of higher degree are gathered in a block, resulting in values of *Q* larger than expected [39]. The small fraction of yellow values below the lower bound approximation also correspond to networks with *B* = 1, but with different (*ξ*, *p*) parameters. In the same spirit, the upper and lower bounds for I can be as well approximated from the actual value of N (see electronic supplementary material, figure S4). For the sake of completeness, *Q*-I scatter plots are shown in electronic supplementary material, figure S5, where we reconfirm that I and *Q* can coexist, i.e. there is no clear map or mutually imposed constraints from one to the other.

The corresponding software codes to obtain the upper and lower bounds for *Q* and I are included in the package that can be downloaded from the web page of the group (http://cosin3.rdi.uoc.edu/), under the Resources section.

## 8. Application to real networks

For the conducted experiments with synthetic networks, we have seen that N provides informative bounds to the mesoscale organization. However, real networks differ from idealized synthetic networks, e.g. the assumption of homogeneous sizes of communities or uncorrelated noise. To assess the accuracy that our development has in real scenarios, we perform experiments on 347 real networks, covering several domains: 57 real unipartite networks [39] (mostly social and economic networks) and 290 bipartite networks (ecological in most cases [59], with some social networks [60] as well).

**Figure 7.**
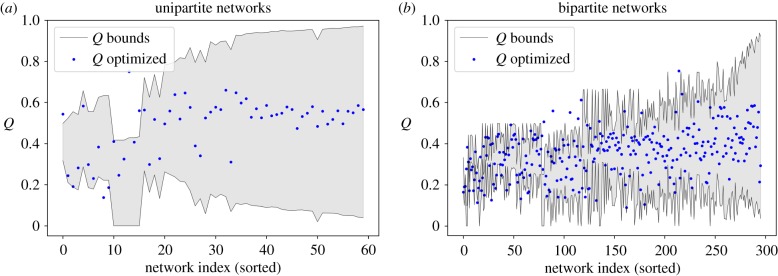
Assessment of the accuracy of the bounds for real networks. (*a*) Corresponds to 57 unipartite networks (figure 6*b*), and (*b*) to 290 bipartite networks (figure 6*c*). In both cases, networks are sorted by the difference between the upper and lower bound. (Online version in colour.)

Remarkably, for these real networks, figure 6*b*,*c*, our bound estimations also hold quite accurately. In general, we observe that for both uni- and bipartite real networks, the limits and bounds for the bipartite case behave as expected. In general, the larger N, the tighter are the bounds of *Q*, and the smaller the maximum value of *Q*. In 45 of these networks the bounds fail: the obtained modularity is either above or below the upper or lower bound, respectively. To ease visualization, figure 7 presents the same results, sorted by the difference between the upper and lower bounds. Results show that, in general, we have a good estimation of the bounds. For the bipartite networks, results are clearer, since higher values of nestedness produce tighter bounds. For the unipartite case, the low values of nestedness of these networks derive wider bounds.

## 9. Discussion

While macro- and mesoscale arrangements in complex networks have been studied in depth, we know little about how they coexist and interfere with each other. Understanding and, above all, quantifying such interferences and possible mutual restrictions, becomes necessary for many reasons. First, because empirical evidence suggests the concurrence of more than one pattern within the same network [29–31,37]. Second, because a preliminary approximation of the mesoscale structural features of a network is appealing, in the face of prohibitive costs to analyse very large amounts of data. Finally, the interplay between different arrangements (prominently nestedness and modularity) is thought fundamental to decipher the dynamical behaviour of many empirical systems (like ecological, economic and technological networks among others). Under this light, a network should not be regarded, for example, as purely modular or purely nested; rather, it may combine structural features that reflect the fact that the system has evolved under different dynamical pressures, favouring different—sometimes competing—arrangements. Indeed, at least in ecology, it has been shown that each structural pattern enables dynamical properties that may be beneficial for an ecosystem in one sense, e.g. increasing species abundances [32,34], but detrimental in another, e.g. diminishing the persistence of species and the system’s resilience [33].

In this work, we have quantified, numerically and analytically, the interference between nestedness (at the macroscale), modularity and in-block nestedness (at the mesoscale) structural organizations, in both uni- and bipartite settings. We show that modularity and nestedness are antagonistic architectures, the growth of one implies the decline of the other, which can be used to estimate mutual bounds in synthetic and real settings. The need to preserve ingredients from both arrangements points at intermediate nested-modular regimes, which are indeed possible with in-block nested structures.

Our results stand as a theoretical and numerical step forward to better understand past empirical evidence, which pointed at the harsh (but not impossible) coexistence of nestedness and modularity, in ecology and elsewhere. In doing so, we pave the way to future research that aims to clarify, from a richer perspective, the role of one or more structural patterns in the assembly and evolution of networked systems. The present paper leaves open three direct lines of development: one, aiming at analytical results for a more general family of networks, particularly for networks with higher connectance (lower *ξ* ) and the inclusion of more realistic settings, such as heterogeneous community size distributions. The second line refers to the dynamical mechanisms that enable the emergence of in-block nested structures, especially from purely nested initial conditions, to reveal to what extent such hybrid architecture is indeed a transitional organization between nestedness and modularity. Finally, in-depth evaluation of the dynamical properties of in-block nested structures is needed, following the rich trail of works that have studied ecologically relevant processes, e.g. abundance maximization and stability.

## Supplementary Material

Electronic Supplementary Material

## Data accessibility

Data and codes used within this article can be downloaded from the web page of the group http://cosin3.rdi.uoc.edu/, under the Resources section and from http://www.web-of-life.es/.
