# Supplementary material for: Macro- and mesoscale pattern interdependencies in complex networks
Source: J R Soc Interface. 2019 Oct 30;16(159):20190553. doi: 10.1098/rsif.2019.0553 (PMC6833316; doi:10.1098/rsif.2019.0553)
Supplement: Electronic Supplementary Material [file rsif20190553supp1.pdf]

Supplemental Material for
"Macro- and mesoscale pattern interdependencies in Complex Networks"

María J. Palazzi[1], Javier Borge-Holthoefer[1], Claudio J. Tessone[2] and Albert Solé-Ribalta[1]
[1]*Internet Interdisciplinary Institute (IN3), Universitat Oberta de Catalunya, Barcelona, Catalonia, Spain*
[2]*URPP Social Networks, Universität Zürich, Switzerland.*
(Dated: September 27, 2019)

I. TERNARY PLOT: DOMINANCE REGIONS

A ternary plot is a three-variable diagram on which each point represents the proportions between three variables. Given the values of the variables, $\mathcal{N}$, $\mathcal{I}$ and Q, the proportions that are eventually represented in the plot are obtained as $f_{\mathcal{N}} = \mathcal{N}^{-1}(\mathcal{N}+\mathcal{I}+Q)$, $f_{\mathcal{I}} = \mathcal{I}^{-1}(\mathcal{N}+\mathcal{I}+Q)$ and $f_Q = Q^{-1}(\mathcal{N}+\mathcal{I}+Q)$. As it was mentioned in the main text, the bottom axis represents $\mathcal{N}$ and its rigth vertex perfectly nested networks ($f_{\mathcal{N}} = 1$). Other values of $f_{\mathcal{N}}$ are indicated by the dashed blue lines in direction $\nearrow$ of the triangle. Rigth axis represent f_Q and the top vertex purely modular networks ($f_Q = 1$). Other f_Q values correspond to horizontal dashed blue lines. Finally, the left axis represents $f_{\mathcal{I}}$ and the left vertex networks that are purely nested ($f_{\mathcal{I}} = 1$). Other $f_{\mathcal{N}}$ values are indicated by lines in direction $\searrow$ of the triangle. Additionally, the black dashed lines delimit dominance regions, which are highlighted in different grey tones for variable pairs in panel a-c and in triads for in panel d. Each dominance region spots (by pairs) which is the dominating structural pattern. For ease of identification the dominant structure is also indicated close to the plot axis. Points over the line of dominance equilibrium in panels a to c correspond to points where the contribution of the two contrasted variables is equivalent.

II. ANALYTIC EXPRESSIONS OF $\mathcal{N}_{G^*}, Q_{G^*}, \mathcal{I}_{G^*}$ ALONG F_2 FOR THE CASES OF $B = 1$ AND $B = 2$

This section provides a complementary formulation for two particular cases of the ring of star graphs, G^*. In the main text, Eqs. 4-6 were developed for the general case $B > 2$. Here, we introduce as well the exact derivation of the expressions $\mathcal{N}_{G^*}$, Q^* and $\mathcal{I}_{G^*}$ for the cases of a single star ($B = 1$) and two stars connected through their central nodes ($B = 2$). As stated in the main text, for these two situations, we have to take into account the change in the number of inter-community links and the degree of the generalist nodes.

A. Nestedness

1. $\mathcal{N}_{G^*}$ for $B = 1$

The computation of the pair overlap for the evaluation of nestedness when $B = 1$ requires only the following terms: the pair overlap of a generalist node (the center of each star subgraph), g, with the specialist nodes s which is $O_{gs}/k_s = 0$; and the

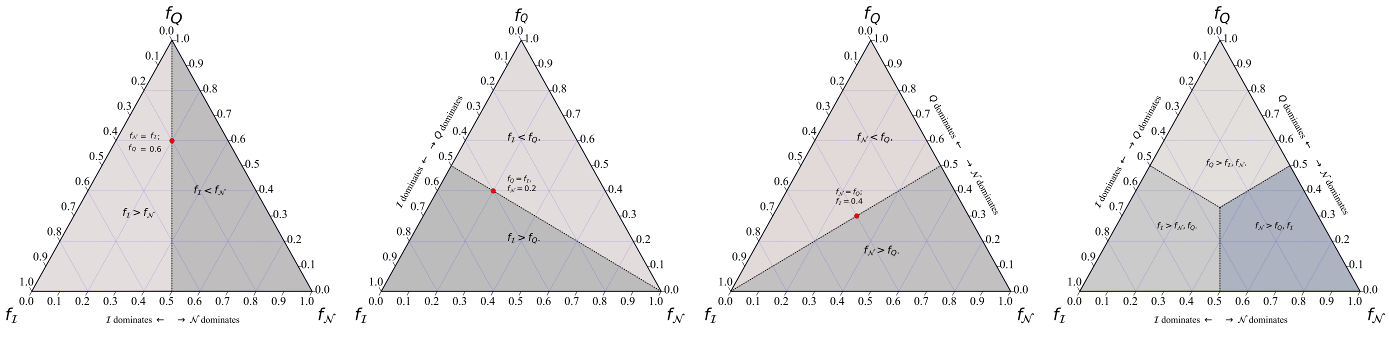

FIG. S1: Representation of the three variables in the ternary plot showing exemplar points with different proportions and evincing the dominance regions. Panel (a) to (c) delimit the dominance regions (in pairs) between nestedness, modularity and in-block nestedness structures. Panel (d) delimits these regions jointly considering all the variables.

pair overlap between all the specialists nodes $O_{ss}/k_s = 1$, the degree of the generalist node is $k_g = N_B - 1$ and the null model corrections $\langle O_{gs} \rangle = k_g k_s / BN_B = (N_B - 1)/BN_B$ and $\langle O_{ss} \rangle = k_s k_s / BN_B = 1/BN_B$

$$\begin{aligned} \mathcal{N}_{G^*} &= \frac{2}{N_B(BN_B - 1)} \left\{ \left[-\frac{N_B - 1}{BN_B}(N_B - 1) \right] + \left[\left(1 - \frac{1}{BN_B} \right) \frac{(N_B - 2)(N_B - 1)}{2} \right] \right\} \\ &= \frac{(N_B - 2)(N_B - 1)}{N_B^2} - \frac{2(N_B - 1)^2}{N_B^2(N_B - 1)}, \end{aligned} \tag{S1}$$

2. $\mathcal{N}_{G^*}$ for $B = 2$

For this scenario we have to take into account the change on the degree of the generalist node $k_g = N_B$ and additional terms such as: the pair overlap between the two generalists O_{gg}/k_g, the pair overlap between a generalist with the specialist from the other community $O_{gs_{out}}/k_s$, and the pair overlap between a specialist with the specialists from the other community$O_{ss_{out}}/k_s$. Finally, we obtain

$$\mathcal{N}_{G^*} = \frac{BN_B^3 - BN_B^2 - 6N_B^2 + 8N_B - 3}{BN_B^2(BN_B - 1)} \tag{S2}$$

B. Modularity

1. Q_{G^*} for $B = 1$

Starting with the equation for modularity expressed as sum over the communities (Eq. 2 of the main text), we obtain the total number of links in the network and the number of links per community for a single star graph $N_B - 1$, and the sum of the degrees of the nodes in the community $d_c = 2(N_B - 1)$. Now, we have the maximum modularity for $B = 1$.

$$Q_{G^*} = B \left[\frac{(N_B - 1)}{(N_B - 1)} - \left(\frac{2(N_B - 1)}{2(N_B - 1)} \right)^2 \right] = 0, \tag{S3}$$

2. Q_{G^*} for $B = 2$

When $B = 2$ the total number of links changes to $L = B(N_B - 1) + 1$ and the sum of the degrees of the nodes in the community is $d_c = 2(N_B - 1) + 1$. So we obtain

$$\begin{aligned} Q_{G^*} &= B \left[\frac{(N_B - 1)}{B(N_B - 1) + 1} - \left(\frac{2N_B - 1}{2(B(N_B - 1) + 1)} \right)^2 \right] \\ &= 2 \left[\frac{(N_B - 1)}{2(N_B - 1) + 1} - \left(\frac{2N_B - 1}{2(2(N_B - 1) + 1)} \right)^2 \right] \\ &= \left[\frac{(2N_B - 2)}{2N_B - 1} - \frac{1}{2} \right], \end{aligned} \tag{S4}$$

C. In-block nestedness

1. $\mathcal{I}_{G^*}$ for $B = 1$

Once again, we know that we will have only two contributing terms to our sum; the pair overlap between specialists (s) nodes and the pair overlap of the generalist (g) node with the specialists. Additionally, we know that for this case the degree of the

generalist node is $k_G = N_B - 1$ and the rest of the terms are: the number of specialists nodes $N_s = (N_B - 1)$, the null model corrections $\langle O_{g,s}\rangle = k_g k_g / BN_B = (N_B - 1)/BN_B$ and $\langle O_{s,s}\rangle = k_s k_s / BN_B = 1/BN_B$, an the size of the communities is $C = N_B$. Finally the analytical expression for in-block nestedness when $B = 1$ reads,

$$\begin{aligned}\mathcal{I}_{G^*} &= \frac{2}{N_B}\left\{\left[\frac{-(N_B-1)/BN_B}{(N_B-1)}(N_B-1)\right]+\left[\frac{1-1/BN_B}{(N_B-1)}\frac{(N_B-2)(N_B-1)}{2}\right]\right\} \\ &= \frac{2}{N_B}\left\{\left[-\frac{(N_B-1)}{N_B}\right]+\left[\frac{(N_B-1)(N_B-2)}{2N_B}\right]\right\},\end{aligned} \tag{S5}$$

2. $\mathcal{I}_{G^*}$ for $B = 2$

Now, for $B = 2$ we have that the degree of the generalist node is $k_G = N_B$. Substituting this term we obtain a new expression for the in-block nestedness as

$$\mathcal{I}_{G^*} = \frac{2}{N_B}\left\{\left[-\frac{1}{N_B}\right]+\left[\frac{(2N_B-1)(N_B-2)}{4N_B}\right]\right\}, \tag{S6}$$

III. ANALYTICAL EXPRESSION FOR BIPARTITE NETWORKS

A. Nestedness

First we shall introduce the bipartite expression for the nestedness measure corrected by a null model.

$$\mathcal{N}_{G^*} = \frac{2}{N_r+N_c}\left\{\sum_{i,j}^{N_r}\left[\frac{O_{i,j}-\langle O_{i,j}\rangle}{k_j(N_r-1)}\Theta(k_i-k_j)\right]+\sum_{l,m}^{N_c}\left[\frac{O_{l,m}-\langle O_{l,m}\rangle}{k_m(N_c-1)}\Theta(k_l-k_m)\right]\right\}, \tag{S7}$$

Now we procced, to present the analytical development: we will have several terms contributing to the sum, regardless of the star they belong to, by effect of the null model. Additionally, we will have pair overlap (PO) between the generalist nodes $(G)_{r,c}$ with all the specialists $(S)_{r,c}$ nodes from its own block $(O_{GS_{in}})$, the PO between the generalist with the specialists of the two blocks he is connected with $(O_{GS_{ring}})$, the contributions of a specialist node with the specialists from its own block $(O_{SS_{in}})$:

$$\begin{aligned}\mathcal{N}_{G^*} &= \frac{2B}{B(N_r+N_c)}\left\{\sum_{i,j}^{N_r}\left[\frac{O_{i,j}-\langle O_{i,j}\rangle}{k_j(BN_r-1)}\Theta(k_i-k_j)\right]+\sum_{l,m}^{N_c}\left[\frac{O_{l,m}-\langle O_{l,m}\rangle}{k_m(BN_c-1)}\Theta(k_l-k_m)\right]\right\} \\ &= \frac{2}{N_r+N_c}\left\{\left[\frac{1-\langle O_{GS_{in}}\rangle}{k_S}N_S\right]+2\left[\frac{1-\langle O_{GS_{ring}}\rangle}{k_S}N_S\right]+\left[\frac{1-\langle O_{GS_{out}}\rangle}{k_S}N_S(B-3)\right]\right. \\ &\quad +2\left[\frac{1-\langle O_{GG_{ring}}\rangle}{k_G}\right]+\left[\frac{1-\langle O_{GG_{out}}\rangle}{k_G}(B-3)\right]+\left[\frac{1-\langle O_{SS_{in}}\rangle}{k_S}(N_S-1)N_S\right] \\ &\quad \left.+\left[\frac{1-\langle O_{SS_{out}}\rangle}{k_S}(N_S)^2(B-1)\right]\right\}_{rows,cols}\end{aligned}$$

where N_S refers to the number of specialists nodes (rows or cols) in the stars communities and is equal to $N_S = (N_c - 1) = (N_r - 1)$, the expressions for the null modell corrections can be generalized to three cases: generalist with specialist $\langle O_{GS}\rangle = k_{G_{r,c}} k_{S_{r,c}}/BN_{r,c} = (N_c+2)/BN_c = (N_r+2)/BN_r$, specialist with specialist $\langle O_{SS}\rangle = k_{S_{r,c}} k_{S_{r,c}}/BN_{r,c} = 1/BN_r = 1/BN_c$, and generalist with generalist $\langle O_{GG}\rangle = k_{G_{r,c}} k_{G_{r,c}}/BN_{r,c} = (N_{r,c}+2)^2/BN_{r,c}$, respectively.

Finally, the expression for the global nestedness $\mathcal{N}$ is,

$$\mathcal{N}_{G^*} = \frac{N_c(N_c+2)(BN_r-1)\left[B\left((N_c^2+N_c-3)N_r^2+N_c^2N_r-2(N_c+2)N_c+N_r+2\right)+(N_r+2)(N_c+N_r+1)\right]+N_r(N_r+2)(BN_c-1)\left[B\left((N_r^2+N_r-3)N_c^2+N_c(N_r^2+1)-2N_r(N_r+2)+2\right)+(N_c+2)(N_c+N_r+1)\right]}{BN_cN_r(N_c+2)(N_c+N_r)(N_r+2)(BN_c-1)(BN_r-1)}$$

B. Modularity

Starting from the expression for modularity in bipartite networks

$$Q_{G^*} = \frac{1}{L}\sum_{i=1}^{N_r}\sum_{j=1}^{N_c}\left(a_{ij} - \frac{k_i^{N_r}k_j^{N_c}}{L}\right)\delta(\alpha_i^{N_r},\alpha_j^{N_c}), \tag{S8}$$

where$L$ the number of links in a, k^{N_r} describes the node degree for row nodes (the number of colum nodes each row node interacts with) and k^{N_c} describes the node degree for colum nodes (the number of row nodes each colum node interacts with), and the Kronecker delta function $\delta(\alpha_i^{N_r},\alpha_j^{N_c})$ that is equal to one when nodes i and j belong to the same module (i.e. they have the same label value) and zero otherwise. Additionally, we can rewrite the expression in terms of the sum over the number of modules

$$Q_{G^*} = \sum_{d=1}^{B}\left[\frac{l_d}{L} - \left(\frac{k_d^r k_d^c}{L^2}\right)\right],$$

where B is the number of communities, l_d is the total number of links within a community d, L is the total number of links in the network, k_d^r is the sum of the degrees of the nodes within module d that belong to nodes r and k_d^c is the sum of the degrees of the nodes within module d that belong to set c. Additionally, in this configuration, we know a priori that the total number of links in the networks is equal to $B(N_r+N_c+1)$[1]; and that number of links per community is equal to N_r+N_c-1. Now, we have the maximum modularity expressed as

$$\begin{aligned} Q_{G^*} &= B\left[\frac{N_r+N_C-1}{B(N_r+N_c+1)} - \left(\frac{(N_r+N_c+1)^r(N_r+N_c+1)^c}{[B(N_r+N_c+1)]^2}\right)\right] \\ &= \frac{N_r+N_c-1}{N_r+N_c+1} - \frac{1}{B} = 1 - \frac{2}{N_r+N_c+1} - \frac{1}{B}, \end{aligned} \tag{S9}$$

For the special case when $B=1$, it can be easily demonstrated that the optimal value of modularity is $Q=0$.

C. In-block nestedness

The expression for the in-block nestedness measure in bipartite networks reads

$$\mathcal{I}_{G^*} = \frac{2}{N_r+N_C}\left\{\sum_{i,j}^{N_r}\left[\frac{O_{i,j}-\langle O_{i,j}\rangle}{k_j(C_i-1)}\Theta(k_i-k_j)\delta(\alpha_i,\alpha_j)\right] + \sum_{l,m}^{N_r}\left[\frac{O_{l,m}-\langle O_{l,m}\rangle}{k_m(C_l-1)}\Theta(k_l-k_m)\delta(\alpha_l,\alpha_m)\right]\right\}, \tag{S10}$$

In this case we will have only two to contributing terms to our sum over rows and cols nodes; the pair overlap between specialists $(S_{r,c})$ nodes and the pair overlap of the generalist $(G_{r,c})$ node with the specialists. Then, we rewrite the expression of Eq. 2.3 as follows,

$$\begin{aligned} \mathcal{I}_{G^*} &= \frac{2B}{B(N_r+N_c)}\left\{\sum_{i,j}^{N_r}\left[\frac{O_{i,j}-\langle O_{i,j}\rangle}{k_j(C_i-1)}\Theta(k_i-k_j)\right] + \sum_{l,m}^{N_c}\left[\frac{O_{l,m}-\langle O_{l,m}\rangle}{k_m(C_l-1)}\Theta(k_l-k_m)\right]\right\} \\ &= \frac{2}{N_r+N_c}\left\{\left[\left(\frac{1-\langle O_{G,S}\rangle}{k_S(C_G-1)}N_S\right) + \left(\frac{1-\langle O_{S,S}\rangle}{k_S(C_S-1)}(N_S-1)N_S\right)\right]_{rows}\right. \\ &\quad \left. + \left[\left(\frac{1-\langle O_{G,S}\rangle}{k_S(C_G-1)}N_S\right) + \left(\frac{1-\langle O_{S,S}\rangle}{k_S(C_S-1)}(N_S-1)N_S\right)\right]_{cols}\right\}, \end{aligned}$$

---

[1] Number of links of all the communities, plus the $2B$ intercommunitiy links $B(N_c+Nr-1)+2B$

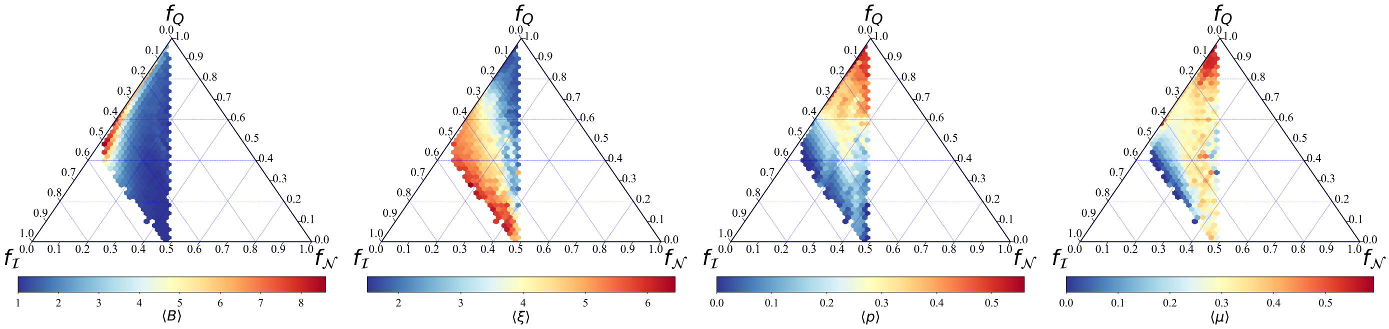

FIG. S2: Ternary plots representing results for $\sim 2\times 10^5$ networks as in the main text. In this case, color in each bin of the simplex indicates the average number of blocks B (a); average shape parameter ξ (b); average intra-block noise p (c); and finally average inter-block noise μ (d).

again N_S refers to the number of specialists nodes (rows or cols) in the stars communities and is equal to $N_S = (N_c-1) = (N_r-1)$, the null modell corrections are equal to $\langle O_{G,S}\rangle = k_G k_S/BN_{r,c} = (N_c+2)/BN_c = (N_r+2)/BN_r$ and $\langle O_{S,S}\rangle = k_S k_S/BN_{r,c} = 1/BN_r = 1/BN_c$, respectively. The size of the communities is $C_G = C_S = N_{r,c}$. Finally the analytical expresion for in-block nestedness reads,

$$\begin{aligned}\mathcal{I}_{G^*} = \frac{2}{N_r+N_c}&\left\{\left[\left(\frac{BN_c-(N_c+2)}{BN_c}\right)+\left(\frac{(BN_c-1)(N_r-2)}{2BN_c}\right)\right]_{rows}\right.\\&\left.+\left[\left(\frac{BN_r-(N_r+2)}{BN_r}\right)+\left(\frac{(BN_r-1)(N_c-2)}{2BN_r}\right)\right]_{cols}\right\}\\&=1-\frac{1}{BN_c}-\frac{1}{BN_r}-\frac{2}{B(N_r+N_c)}-\frac{2}{BN_rN_c},\end{aligned} \tag{S11}$$

IV. NESTEDNESS, MODULARITY AND IN-BLOCK NESTEDNESS IN LIMITING CASES: BIPARTITE EXPRESSIONS

These correspond to scenarios in which the number of blocks, B, and the size of the blocks, N_B, tend to ∞.
We start with $N_B\to\infty$. In this case, Eqs. III A and S9 reduce to

$$\lim_{N_{r,c}\to\infty}\mathcal{N}_{G^*} = \frac{1}{B}, \qquad \lim_{N_{r,c}\to\infty}Q_{G^*} = 1-\frac{1}{B}, \tag{S12}$$

With respect to the case $B\to\infty$, Eqs. III A and S9 turn now

$$\lim_{B\to\infty}\mathcal{N}_{G^*} = 0, \qquad \lim_{B\to\infty}Q_{G^*} = 1-\frac{2}{N_r+N_c+1}. \tag{S13}$$

Finally, with respect to in-block nestedness, the analytical calculations in both limits yield

$$\lim_{N_{r,c}\to\infty}\mathcal{I}_{G^*} = 1, \qquad \lim_{B\to\infty}\mathcal{I}_{G^*} = 1 \tag{S14}$$

V. SUPPLEMENTAL FIGURES 2 AND 3

Figure S2 shows the results with respect to the parameters of the probabilistic network generation model employed to perform the numerical exploration as explained in the main text (Fig. 1). The model is described in detail in the Appendix A of the main text. Panels (a)-(d) show the results with respect to varying values of the number of blocks (B), shape parameter (ξ), intra-block (p) and inter-block noise (μ), respectively. The colour bar indicates the mean value of the respective parameter in each bin of the simplex.

Figure S3 explains the decision, stated in Section 4 of the main text, to restrict the levels of intra- and inter-block noise (i.e. $p\le 0.6$, $\mu\le 0.6$) in the synthetic benchmark. We wanted to introduce a considerable level of noise while guaranteeing that some

identifiable pattern was still present. To this aim, we have followed the concept of weak modularity as introduced in Radicchi *et al.* (ref. [15] of the main text). In that work, authors define weak modularity as a network partition in which modules have more internal than external links, but that is not true for each and every node in those modules. This informal notion guided our limitation of μ noise: we stop generating networks beyond $\mu = 0.6$ because the imposed community structure does not even comply with the weak modularity condition (and thus an algorithm could hardly detect it). To prove this point, we show in Fig. S3 that, for $\mu = 0.55$, still $\sim 65\%$ of the generated networks are weakly modular; by the time $\mu = 0.6$, only 40% of them fulfils the condition.

It should also be noted that, even completely random networks exhibit a remarkable level of modularity Q [1]. Since, by definition, $\mathcal{I} = \mathcal{N} = 0$ in such situation, any completely random realisation of the benchmark will induce a (false) modular-dominant network in the ternary plot –as, indeed, it already happens for high levels of p and μ, see the corresponding panels in Fig. S2

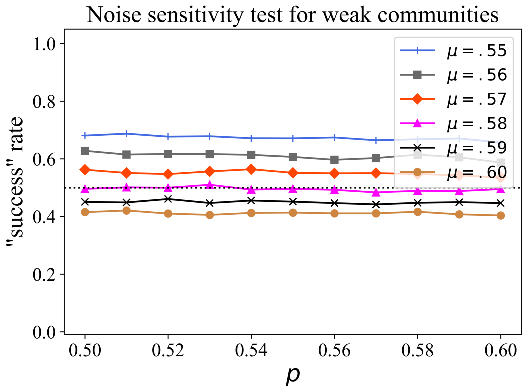

FIG. S3: Noise sensitivity test: Rate of networks that fulfill the condition for weak communities for different combinations of the noise parameters p and μ.

VI. SUPPLEMENTAL FIGURE 4

Figure S4 shows the values of Q plotted against $\mathcal{N}$, for the all the generated networks employed in the numerical exploration in the main text ($\sim 2 \times 10^5$). The corresponding upper and lower bounds were plotted on top. The color bar, in each case, indicates the values of the respective parameters of the probabilistic network generation model (number of blocks (B), shape parameter (ξ), intra-block (p) and inter-block noise (μ), respectively).

VII. SUPPLEMENTAL FIGURES 5 AND 6

For the sake of completeness, we have plotted the values of $\mathcal{I}$ against $\mathcal{N}$ (Fig. S5) and Q against $\mathcal{I}$ (Fig. S6). Similar to Fig. 3A in the main text, upper and lower bounds for $\mathcal{I}$ have been calculated in Fig. S5, taking actual measurements of $\mathcal{N}$ as a

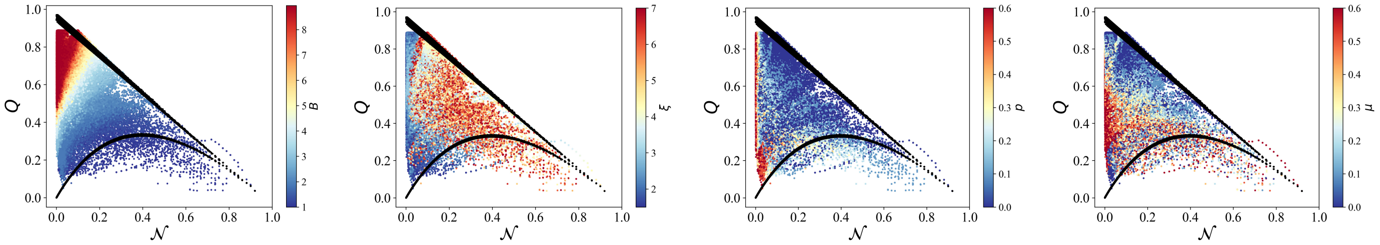

FIG. S4: Optimized values of Q plotted against $\mathcal{N}$, for the generated networks. The values of the corresponding upper and lower bounds were plotted on top (black dots). The color bar indicates the value of the respective parameters of the probabilistic network generation model (number of blocks (B), shape parameter (ξ), intra-block (p) and inter-block noise (μ), respectively).

starting point. Remarkably, none of the optimized values of $\mathcal{I}$ violates such bounds. This is no surprise with respect to lower bounds, since $\mathcal{I}$ reduces to $\mathcal{N}$ when $B = 1$, thus the lower bound simply represents the hard limit $\mathcal{I} = \mathcal{N}$. But even the upper bounds, which represent an estimation, are in excellent agreement with respect to the optimized values of $\mathcal{I}$.

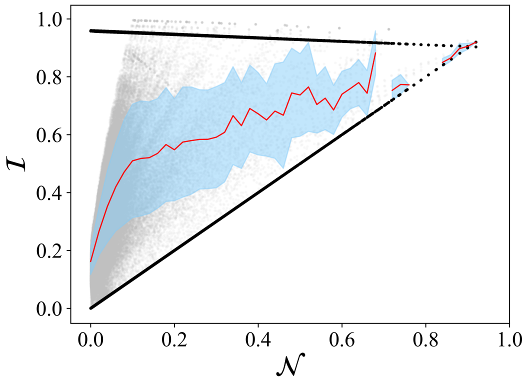

FIG. S5: Values of $\mathcal{I}$ obtained after optimization (grey dots), plotted against $\mathcal{N}$ for the generated networks. Upper and lower bounds of $\mathcal{I}$ are plotted in colors. The color bar indicates the network's size

On the other hand, the main lesson from Fig. S6 is the fact that, unlike Q and $\mathcal{N}$, other patterns can coexist, i.e. there is no clear map between Q and $\mathcal{I}$.

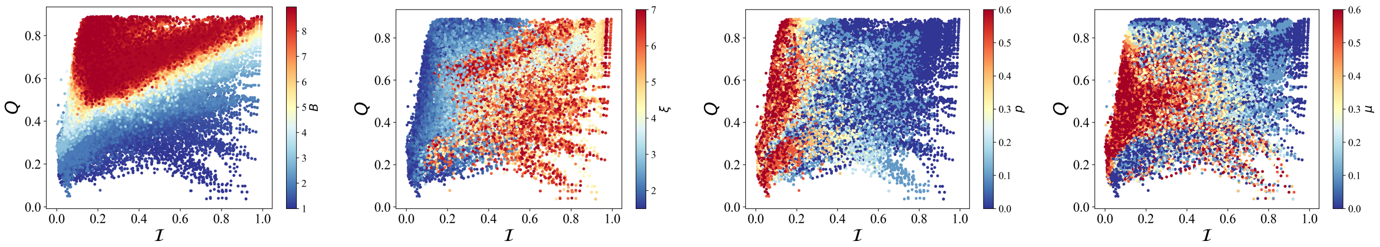

FIG. S6: Optimized values of Q plotted against the optimized values of $\mathcal{I}$, for the generated networks. The color bar indicates the value of the respective parameters of the probabilistic network generation model. Panel (a) shows the results with respect to the number of blocks. Panel (b) corresponds to the shape parameter ξ. Panels (c) and (d) corresponds to the noise parameters p and μ, respectively.

VIII. REAL DATASETS USED IN THE EXPERIMENTS

Te following section presents the set of real networks used throughout the article comprise ecological and social systems. The largest subset – ecological networks [9] – represent mutualistic and competitive systems. The analysed social systems include social communication networks such as face-to-face interactions, e- mail contacts [6, 8] and cooperative software development projects [10]. The data regarding the cooperative software development projects, will be availble at the web page of the group `http://cosin3.rdi.uoc.edu/`, under the Resources section.

ID	Especies	Type	Relation Type
A_HP_001	28	Bipartite	Host-Parasite
A_HP_002	42	Bipartite	Host-Parasite
A_HP_003	32	Bipartite	Host-Parasite
A_HP_004	27	Bipartite	Host-Parasite
A_HP_005	20	Bipartite	Host-Parasite
A_HP_006	53	Bipartite	Host-Parasite
A_HP_007	25	Bipartite	Host-Parasite
A_HP_008	32	Bipartite	Host-Parasite
A_HP_009	36	Bipartite	Host-Parasite
A_HP_010	49	Bipartite	Host-Parasite

ID	Especies	Type	Relation Type
A_HP_011	23	Bipartite	Host-Parasite
A_HP_012	30	Bipartite	Host-Parasite
A_HP_013	33	Bipartite	Host-Parasite
A_HP_014	30	Bipartite	Host-Parasite
A_HP_015	10	Bipartite	Host-Parasite
A_HP_016	27	Bipartite	Host-Parasite
A_HP_017	14	Bipartite	Host-Parasite
A_HP_018	36	Bipartite	Host-Parasite
A_HP_019	27	Bipartite	Host-Parasite
A_HP_020	33	Bipartite	Host-Parasite

ID	Especies	Type	Relation Type
A_HP_021	24	Bipartite	Host-Parasite
A_HP_022	34	Bipartite	Host-Parasite
A_HP_023	17	Bipartite	Host-Parasite
A_HP_024	27	Bipartite	Host-Parasite
A_HP_025	58	Bipartite	Host-Parasite
A_HP_026	33	Bipartite	Host-Parasite
A_HP_027	47	Bipartite	Host-Parasite
A_HP_028	19	Bipartite	Host-Parasite
A_HP_029	49	Bipartite	Host-Parasite
A_HP_030	29	Bipartite	Host-Parasite
A_HP_031	56	Bipartite	Host-Parasite
A_HP_032	27	Bipartite	Host-Parasite
A_HP_033	47	Bipartite	Host-Parasite
A_HP_034	17	Bipartite	Host-Parasite
A_HP_035	13	Bipartite	Host-Parasite
A_HP_036	21	Bipartite	Host-Parasite
A_HP_037	38	Bipartite	Host-Parasite
A_HP_038	30	Bipartite	Host-Parasite
A_HP_039	17	Bipartite	Host-Parasite
A_HP_040	26	Bipartite	Host-Parasite
A_HP_041	21	Bipartite	Host-Parasite
A_HP_042	53	Bipartite	Host-Parasite
A_HP_043	38	Bipartite	Host-Parasite
A_HP_044	53	Bipartite	Host-Parasite
A_HP_045	23	Bipartite	Host-Parasite
A_HP_046	56	Bipartite	Host-Parasite
A_HP_047	37	Bipartite	Host-Parasite
A_HP_048	26	Bipartite	Host-Parasite
A_HP_049	24	Bipartite	Host-Parasite
A_HP_050	62	Bipartite	Host-Parasite
A_HP_051	39	Bipartite	Host-Parasite
A_PH_004	74	Bipartite	Plant-Herbivore
A_PH_005	78	Bipartite	Plant-Herbivore
A_PH_006	94	Bipartite	Plant-Herbivore
A_PH_007	69	Bipartite	Plant-Herbivore
M_PA_001	24	Bipartite	Plant-Ant
M_PA_002	10	Bipartite	Plant-Ant
M_PA_003	39	Bipartite	Plant-Ant
M_PA_004	89	Bipartite	Plant-Ant
M_PL_001	185	Bipartite	Pollination
M_PL_002	107	Bipartite	Pollination
M_PL_003	61	Bipartite	Pollination
M_PL_004	114	Bipartite	Pollination
M_PL_005	371	Bipartite	Pollination
M_PL_006	78	Bipartite	Pollination
M_PL_007	52	Bipartite	Pollination
M_PL_008	49	Bipartite	Pollination
M_PL_009	142	Bipartite	Pollination
M_PL_010	107	Bipartite	Pollination
M_PL_011	27	Bipartite	Pollination
M_PL_012	84	Bipartite	Pollination
M_PL_013	65	Bipartite	Pollination
M_PL_014	110	Bipartite	Pollination
M_PL_015	797	Bipartite	Pollination
M_PL_016	205	Bipartite	Pollination
M_PL_017	104	Bipartite	Pollination
M_PL_018	144	Bipartite	Pollination
M_PL_019	125	Bipartite	Pollination
M_PL_020	111	Bipartite	Pollination
M_PL_021	768	Bipartite	Pollination
M_PL_022	66	Bipartite	Pollination
M_PL_023	95	Bipartite	Pollination
M_PL_024	29	Bipartite	Pollination

ID	Especies	Type	Relation Type
M_PL_025	57	Bipartite	Pollination
M_PL_026	159	Bipartite	Pollination
M_PL_027	78	Bipartite	Pollination
M_PL_028	180	Bipartite	Pollination
M_PL_029	167	Bipartite	Pollination
M_PL_030	81	Bipartite	Pollination
M_PL_031	97	Bipartite	Pollination
M_PL_032	40	Bipartite	Pollination
M_PL_033	47	Bipartite	Pollination
M_PL_034	154	Bipartite	Pollination
M_PL_035	97	Bipartite	Pollination
M_PL_036	22	Bipartite	Pollination
M_PL_037	50	Bipartite	Pollination
M_PL_038	50	Bipartite	Pollination
M_PL_039	68	Bipartite	Pollination
M_PL_040	72	Bipartite	Pollination
M_PL_041	74	Bipartite	Pollination
M_PL_042	18	Bipartite	Pollination
M_PL_043	110	Bipartite	Pollination
M_PL_044	719	Bipartite	Pollination
M_PL_045	43	Bipartite	Pollination
M_PL_046	60	Bipartite	Pollination
M_PL_047	205	Bipartite	Pollination
M_PL_048	266	Bipartite	Pollination
M_PL_049	262	Bipartite	Pollination
M_PL_050	49	Bipartite	Pollination
M_PL_051	104	Bipartite	Pollination
M_PL_052	54	Bipartite	Pollination
M_PL_053	393	Bipartite	Pollination
M_PL_054	431	Bipartite	Pollination
M_PL_055	259	Bipartite	Pollination
M_PL_056	456	Bipartite	Pollination
M_PL_057	997	Bipartite	Pollination
M_PL_058	113	Bipartite	Pollination
M_PL_059	26	Bipartite	Pollination
M_PL_060_01	50	Bipartite	Pollination
M_PL_060_02	50	Bipartite	Pollination
M_PL_060_03	58	Bipartite	Pollination
M_PL_060_04	67	Bipartite	Pollination
M_PL_060_05	87	Bipartite	Pollination
M_PL_060_06	71	Bipartite	Pollination
M_PL_060_07	68	Bipartite	Pollination
M_PL_060_08	47	Bipartite	Pollination
M_PL_060_09	58	Bipartite	Pollination
M_PL_060_10	39	Bipartite	Pollination
M_PL_060_11	34	Bipartite	Pollination
M_PL_060_12	37	Bipartite	Pollination
M_PL_060_13	38	Bipartite	Pollination
M_PL_060_14	48	Bipartite	Pollination
M_PL_060_15	51	Bipartite	Pollination
M_PL_060_16	56	Bipartite	Pollination
M_PL_060_17	52	Bipartite	Pollination
M_PL_060_18	48	Bipartite	Pollination
M_PL_060_19	31	Bipartite	Pollination
M_PL_060_20	30	Bipartite	Pollination
M_PL_060_21	28	Bipartite	Pollination
M_PL_060_22	44	Bipartite	Pollination
M_PL_060_23	39	Bipartite	Pollination
M_PL_060_24	38	Bipartite	Pollination
M_PL_061_01	17	Bipartite	Pollination
M_PL_061_02	22	Bipartite	Pollination
M_PL_061_03	20	Bipartite	Pollination
M_PL_061_04	22	Bipartite	Pollination

ID	Especies	Type	Relation Type
M_PL_061_05	34	Bipartite	Pollination
M_PL_061_06	35	Bipartite	Pollination
M_PL_061_07	32	Bipartite	Pollination
M_PL_061_08	25	Bipartite	Pollination
M_PL_061_09	16	Bipartite	Pollination
M_PL_061_10	19	Bipartite	Pollination
M_PL_061_11	18	Bipartite	Pollination
M_PL_061_12	19	Bipartite	Pollination
M_PL_061_13	26	Bipartite	Pollination
M_PL_061_14	17	Bipartite	Pollination
M_PL_061_15	24	Bipartite	Pollination
M_PL_061_16	27	Bipartite	Pollination
M_PL_061_17	22	Bipartite	Pollination
M_PL_061_18	29	Bipartite	Pollination
M_PL_061_19	32	Bipartite	Pollination
M_PL_061_20	25	Bipartite	Pollination
M_PL_061_21	29	Bipartite	Pollination
M_PL_061_22	28	Bipartite	Pollination
M_PL_061_23	36	Bipartite	Pollination
M_PL_061_24	29	Bipartite	Pollination
M_PL_061_25	16	Bipartite	Pollination
M_PL_061_26	20	Bipartite	Pollination
M_PL_061_27	19	Bipartite	Pollination
M_PL_061_28	27	Bipartite	Pollination
M_PL_061_29	26	Bipartite	Pollination
M_PL_061_30	20	Bipartite	Pollination
M_PL_061_31	24	Bipartite	Pollination
M_PL_061_32	24	Bipartite	Pollination
M_PL_061_33	8	Bipartite	Pollination
M_PL_061_34	10	Bipartite	Pollination
M_PL_061_35	22	Bipartite	Pollination
M_PL_061_36	24	Bipartite	Pollination
M_PL_061_37	30	Bipartite	Pollination
M_PL_061_38	33	Bipartite	Pollination
M_PL_061_39	30	Bipartite	Pollination
M_PL_061_40	35	Bipartite	Pollination
M_PL_061_41	13	Bipartite	Pollination
M_PL_061_42	17	Bipartite	Pollination
M_PL_061_43	25	Bipartite	Pollination
M_PL_061_44	21	Bipartite	Pollination
M_PL_061_45	34	Bipartite	Pollination
M_PL_061_46	34	Bipartite	Pollination
M_PL_061_47	35	Bipartite	Pollination
M_PL_061_48	23	Bipartite	Pollination
M_PL_062	1500	Bipartite	Pollination
M_PL_063	64	Bipartite	Pollination
M_PL_064	22	Bipartite	Pollination
M_PL_065	26	Bipartite	Pollination
M_PL_066	36	Bipartite	Pollination
M_PL_067	36	Bipartite	Pollination
M_PL_068	40	Bipartite	Pollination
M_PL_069_01	24	Bipartite	Pollination
M_PL_069_02	14	Bipartite	Pollination
M_PL_069_03	11	Bipartite	Pollination
M_PL_070	16	Bipartite	Pollination
M_PL_071	52	Bipartite	Pollination
M_SD_001	28	Bipartite	Seed-Dispersal
M_SD_002	40	Bipartite	Seed-Dispersal
M_SD_003	41	Bipartite	Seed-Dispersal
M_SD_004	54	Bipartite	Seed-Dispersal
M_SD_005	38	Bipartite	Seed-Dispersal
M_SD_006	36	Bipartite	Seed-Dispersal
M_SD_007	79	Bipartite	Seed-Dispersal

ID	Especies	Type	Relation Type
M_SD_008	26	Bipartite	Seed-Dispersal
M_SD_009	25	Bipartite	Seed-Dispersal
M_SD_010	64	Bipartite	Seed-Dispersal
M_SD_011	25	Bipartite	Seed-Dispersal
M_SD_012	64	Bipartite	Seed-Dispersal
M_SD_013	55	Bipartite	Seed-Dispersal
M_SD_014	33	Bipartite	Seed-Dispersal
M_SD_015	32	Bipartite	Seed-Dispersal
M_SD_016	85	Bipartite	Seed-Dispersal
M_SD_017	24	Bipartite	Seed-Dispersal
M_SD_018	61	Bipartite	Seed-Dispersal
M_SD_019	209	Bipartite	Seed-Dispersal
M_SD_020	58	Bipartite	Seed-Dispersal
M_SD_021	46	Bipartite	Seed-Dispersal
M_SD_022	317	Bipartite	Seed-Dispersal
M_SD_023	23	Bipartite	Seed-Dispersal
M_SD_024	19	Bipartite	Seed-Dispersal
M_SD_025	13	Bipartite	Seed-Dispersal
M_SD_026	6	Bipartite	Seed-Dispersal
M_SD_027	16	Bipartite	Seed-Dispersal
M_SD_028	13	Bipartite	Seed-Dispersal
M_SD_029	9	Bipartite	Seed-Dispersal
M_SD_030	9	Bipartite	Seed-Dispersal
M_SD_031	49	Bipartite	Seed-Dispersal
M_SD_032	23	Bipartite	Seed-Dispersal
M_SD_033	24	Bipartite	Seed-Dispersal
M_SD_034	121	Bipartite	Seed-Dispersal
AFNetworking	924	Bipartite	Github Project [10]
Alamofire	284	Bipartite	Github Project [10]
Apollo-11	472	Bipartite	Github Project [10]
Chart.js	423	Bipartite	Github Project [10]
Font-Awesome	4971	Bipartite	Github Project [10]
FreeCodeCamp	3349	Bipartite	Github Project [10]
Ghost	3528	Bipartite	Github Project [10]
Modernizr	12764	Bipartite	Github Project [10]
N1	8418	Bipartite	Github Project [10]
RxJava	10475	Bipartite	Github Project [10]
angular.js	4647	Bipartite	Github Project [10]
angular	7264	Bipartite	Github Project [10]
animate.css	331	Bipartite	Github Project [10]
ansible	5847	Bipartite	Github Project [10]
async	1462	Bipartite	Github Project [10]
atom	10924	Bipartite	Github Project [10]
backbone	583	Bipartite	Github Project [10]
bootstrap	4094	Bipartite	Github Project [10]
brackets	7876	Bipartite	Github Project [10]
chosen	220	Bipartite	Github Project [10]
d3	2257	Bipartite	Github Project [10]
django	11847	Bipartite	Github Project [10]
docker	10704	Bipartite	Github Project [10]
electron	2751	Bipartite	Github Project [10]
express	1095	Bipartite	Github Project [10]
flask	823	Bipartite	Github Project [10]
foundation-sites	13382	Bipartite	Github Project [10]
gitignore	1086	Bipartite	Github Project [10]
hackathon-starter	1025	Bipartite	Github Project [10]
hacker-scripts	94	Bipartite	Github Project [10]
html5-boilerplate	622	Bipartite	Github Project [10]
httpie	169	Bipartite	Github Project [10]
impress.js	88	Bipartite	Github Project [10]
ionic	7544	Bipartite	Github Project [10]
jQuery-File-Upload	207	Bipartite	Github Project [10]
javascript	355	Bipartite	Github Project [10]

ID	Especies	Type	Relation Type
jekyll	1879	Bipartite	Github Project [10]
jquery	963	Bipartite	Github Project [10]
laravel	2033	Bipartite	Github Project [10]
lodash	4011	Bipartite	Github Project [10]
material-design-lite	5132	Bipartite	Github Project [10]
material-ui	7072	Bipartite	Github Project [10]
materialize	816	Bipartite	Github Project [10]
meteor	6126	Bipartite	Github Project [10]
moment	1593	Bipartite	Github Project [10]
neovim	4105	Bipartite	Github Project [10]
nw.js	3337	Bipartite	Github Project [10]
oh-my-zsh	1864	Bipartite	Github Project [10]
react-native	10374	Bipartite	Github Project [10]
react	4249	Bipartite	Github Project [10]
redis	1333	Bipartite	Github Project [10]
redux	1261	Bipartite	Github Project [10]
requests	813	Bipartite	Github Project [10]
resume.github.com	82	Bipartite	Github Project [10]
reveal.js	414	Bipartite	Github Project [10]
select2	2015	Bipartite	Github Project [10]
socket.io	1222	Bipartite	Github Project [10]
thefuck	375	Bipartite	Github Project [10]
three.js	7671	Bipartite	Github Project [10]
todomvc	13267	Bipartite	Github Project [10]
underscore	347	Bipartite	Github Project [10]
vscode	8352	Bipartite	Github Project [10]
vue	1264	Bipartite	Github Project [10]
webpack	2043	Bipartite	Github Project [10]
youtube-dl	1526	Bipartite	Github Project [10]
bunt0	31	unipartite	Social contacts [2]
bunt1	32	unipartite	Social contacts [2]
bunt3	32	unipartite	Social contacts [2]
bunt4	32	unipartite	Social contacts [2]
bunt5	32	unipartite	Social contacts [2]
bunt6	32	unipartite	Social contacts [2]
c1	26	unipartite	Social contacts [3]
c2	26	unipartite	Social contacts [3]
c3	26	unipartite	Social contacts [3]
c4	26	unipartite	Social contacts [3]
E-mail contacts (Milchaski)	196	unipartite	Social contacts
stu98t0	140	unipartite	Social contacts [2]
stu98t2	288	unipartite	Social contacts [2]
stu98t3	384	unipartite	Social contacts [2]
stu98t5	421	unipartite	Social contacts [2]
stu98t6	287	unipartite	Social contacts [2]
Primary School	368	unipartite	Social contacts [4]
High School 2011	419	unipartite	Social contacts [5]
High School 2012	1053	unipartite	Social contacts [5]
E-mail contacts	1286	unipartite	Social [6]
zachary	2486	unipartite	Social [7]
Enron E-mail contacts M12	2478	unipartite	Social [8]
Enron E-mail contacts M13	2082	unipartite	Social [8]
Enron E-mail contacts M14	2159	unipartite	Social [8]
Enron E-mail contacts M15	3107	unipartite	Social [8]
Enron E-mail contacts M16	3480	unipartite	Social [8]
Enron E-mail contacts M17	3492	unipartite	Social [8]
Enron E-mail contacts M18	3991	unipartite	Social [8]
Enron E-mail contacts M19	4292	unipartite	Social [8]
Enron E-mail contacts M20	5139	unipartite	Social [8]
Enron E-mail contacts M21	4794	unipartite	Social [8]
Enron E-mail contacts M22	4082	unipartite	Social [8]
Enron E-mail contacts M23	3811	unipartite	Social [8]
Enron E-mail contacts M24	4342	unipartite	Social [8]

ID	Especies	Type	Relation Type
Enron E-mail contacts M25	4326	unipartite	Social [8]
Enron E-mail contacts M26	6338	unipartite	Social [8]
Enron E-mail contacts M27	7288	unipartite	Social [8]
Enron E-mail contacts M28	5014	unipartite	Social [8]
Enron E-mail contacts M29	4585	unipartite	Social [8]
Enron E-mail contacts M30	4703	unipartite	Social [8]
Enron E-mail contacts M31	6743	unipartite	Social [8]
Enron E-mail contacts M32	7727	unipartite	Social [8]
Enron E-mail contacts M33	6736	unipartite	Social [8]
Enron E-mail contacts M34	2975	unipartite	Social [8]
Enron E-mail contacts M35	3110	unipartite	Social [8]
Enron E-mail contacts M36	2232	unipartite	Social [8]
Enron E-mail contacts M37	1640	unipartite	Social [8]
Enron E-mail contacts M38	314	unipartite	Social [8]
Enron E-mail contacts M39	1029	unipartite	Social [8]
Enron E-mail contacts M40	307	unipartite	Social [8]
Enron E-mail contacts M41	126	unipartite	Social [8]
Enron E-mail contacts M42	180	unipartite	Social [8]
Enron E-mail contacts M43	242	unipartite	Social [8]
Enron E-mail contacts M44	32	unipartite	Social [8]
Enron E-mail contacts M45	34	unipartite	Social [8]
Enron E-mail contacts M46	34	unipartite	Social [8]
Enron E-mail contacts M47	34	unipartite	Social [8]
Enron E-mail contacts M48	34	unipartite	Social [8]
Enron E-mail contacts M49	1133	unipartite	Social [8]
Enron E-mail contacts M50	34	unipartite	Social [8]

[1] Guimerá R, Sales-Pardo M and Amaral LAN. *Physical Review E.* 70, 025101. 2004 in Open Source Software, *arXiv preprint*, arXiv:1903.03375.
[2] G. G. Van de Bunt, M. A. Van Duijn, and T. A. Snijders, *Computational & Mathematical Organization Theory* 5, 167. 1999.
[3] T. A. Snijders, C. E. Steglich, and G. G. van de Bunt, *Social Networks* 2008.
[4] J. Stehlé, N. Voirin, A. Barrat, C. Cattuto, L. Isella, J.-F. Pinton, M. Quaggiotto, W. Van den Broeck, C. Regis, B. Lina, et al., *PloS one* 6, e23176, 2011.
[5] J. Fournet and A. Barrat, *PloS one* 9, e107878, 2014.
[6] R. Guimerá, L. Danon, A. Diaz-Guilera, F. Giralt, and A. Arenas, *Physical Review E* 68, 065103, 2003.
[7] W. W. Zachary, *Journal of anthropological research* 33, 452, 1977.
[8] B. Klimt and Y. Yang, *Proc. Eur. Conf. Machine learning (ECML)* , 217, 2004.
[9] `http://www.web-of-life.es/`
[10] M. J .Palazzi, J. Cabot, J. L. Cánovas Izquierdo, A Solé-Ribalta and J. Borge-Holthoefer. Online division of labour: emergent structures
